# Retraction: Machine vision model for drip leakage detection of pipeline

**DOI:** 10.1371/journal.pone.0337862

**Published:** 2025-12-01

**Authors:** The PLOS One Editors

After this article [1] was published, PLOS received information indicating that the listed corresponding author, YW, was included in the author list of [1] without their knowledge or consent, that YW was not involved in the research reported in [1], and that the email address listed in [1] for YW does not belong to them.

YW requested the article’s retraction.

PLOS have been unable to verify the contact emails provided for authors YZ or GZ and have not received responses regarding this matter from YZ, GZ, or the institution where the research was conducted.

In light of the above concerns, and since PLOS has not received the information needed to verify the article’s reliability, the *PLOS One* Editors retract this article.

At the time of retraction, [1] was republished to remove YW from the author byline and to update CX’s affiliation.

CX did not agree with the retraction. YZ and GZ either did not respond directly or could not be reached.
